# Supplementary material for: How Do Molecular Tweezers Bind to Proteins? Lessons from X-ray Crystallography
Source: Molecules. 2024 Apr 12;29(8):1764. doi: 10.3390/molecules29081764 (PMC11051928; doi:10.3390/molecules29081764)
Supplement: Supplementary file 1 [file molecules-29-01764-s001.zip › molecules-2908888-supplementary.pdf]

How Do Molecular Tweezers Bind to Proteins? Lessons from X-ray Crystallography - Supplementary figures

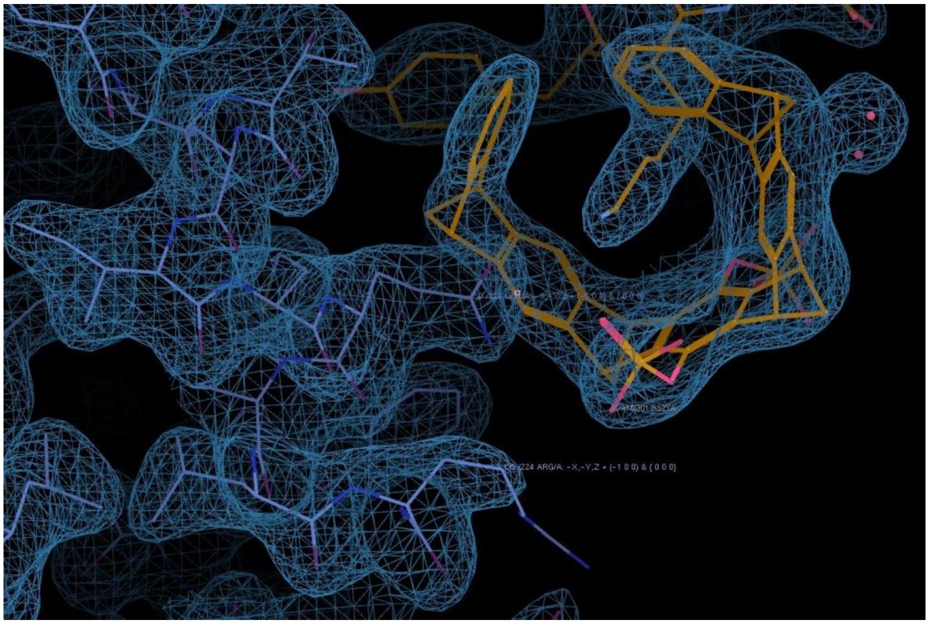

Supplementary Figure S1:

Electron density of the tweezer in the 14-3-3 structure 5OEH (yellow) bound to Lys214 and "clamped" by a symmetry-related molecule (blue)

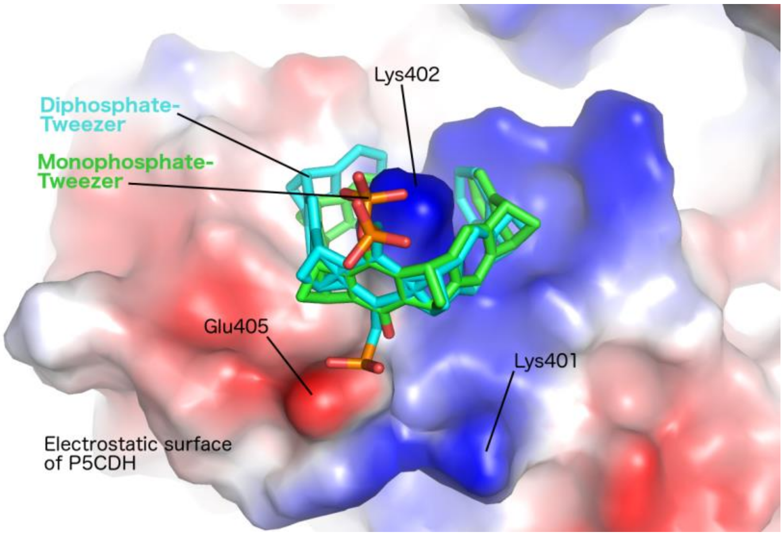

Supplementary Figure S2:

The superimposition of a putative diphosphate-tweezer onto the crystal structure of the monophosphate-tweezer bound to P5CDH shows a potential clash of the second phosphate group with the protein surface next to Glu405. In addition, the negatively charged surface close to this phosphate group would result in electrostatic repulsion.

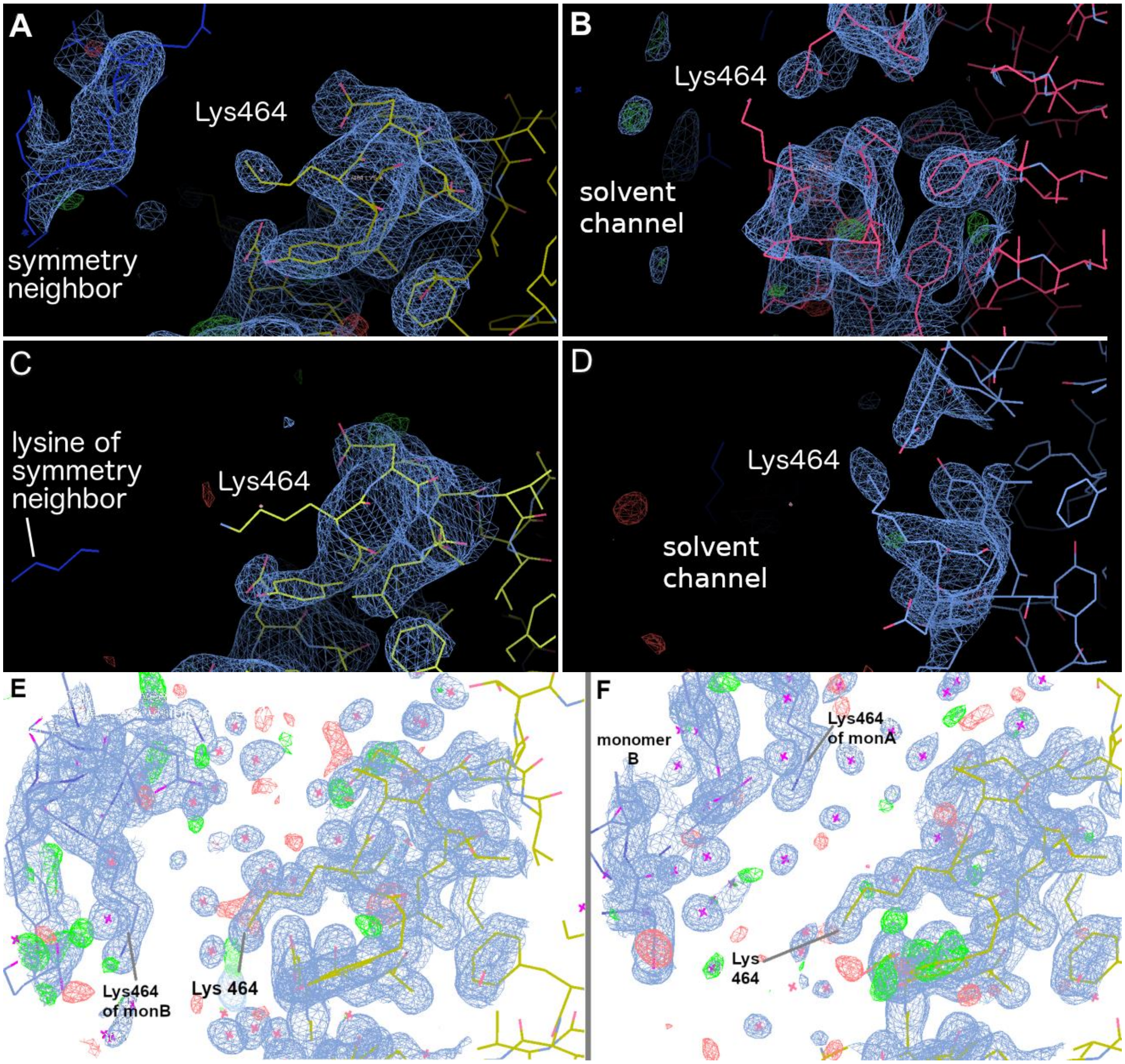

Supplementary Figure S3:

Crystal environment of Lys464 of the P5CDH-diphosphate-tweezer (A-D) and P5CDH-monophosphate-tweezer complexes (E,F). The four monomers of the $P6_5$ crystal form in (A-D) show Lys464 pointing into a solvent channel, so there would be plenty of space for a tweezer molecule in principle in at least three (B,C,D) of the four monomers. However, the conformations of the neighboring side chains seem to be unfavourable for tweezer binding since no electron density corresponding to a tweezer molecule can be observed.

In contrast, in the two monomers of the $P2_1$ crystal form of the P5CDH-monophosphate-tweezer complex, Lys464 is located in a narrow solvent channel (E,F) so there would not be sufficient space for a tweezer molecule. In addition, a symmetry-related Lys464 is nearby so that the two putatively bound tweezers would clash with each other.

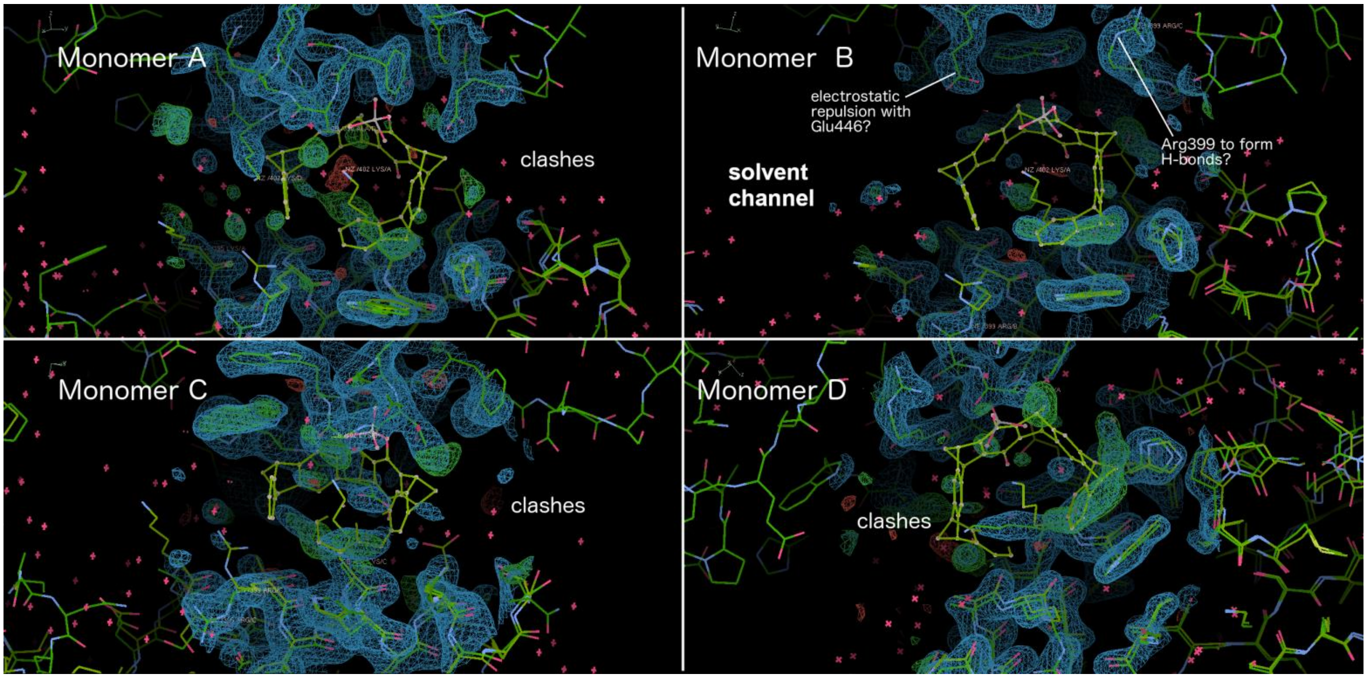

Supplementary Figure S4:

Environment of Lys402 in the $P2_1$ crystal form of apo-P5CDH (PDB ID 4OE5) superimposed with monomer A of the P5CDH-monophosphate-tweezer-complex shows that this crystal form would not be compatible with tweezer binding. The crystal packing excludes binding to Lys402 in at least three of four monomers. It potentially allows binding of the tweezer molecule to Lys402 of monomer B of the asymmetric unit, but the tweezer might have to compete with non-covalently bound ligands as indicated by the overlapping electron density.

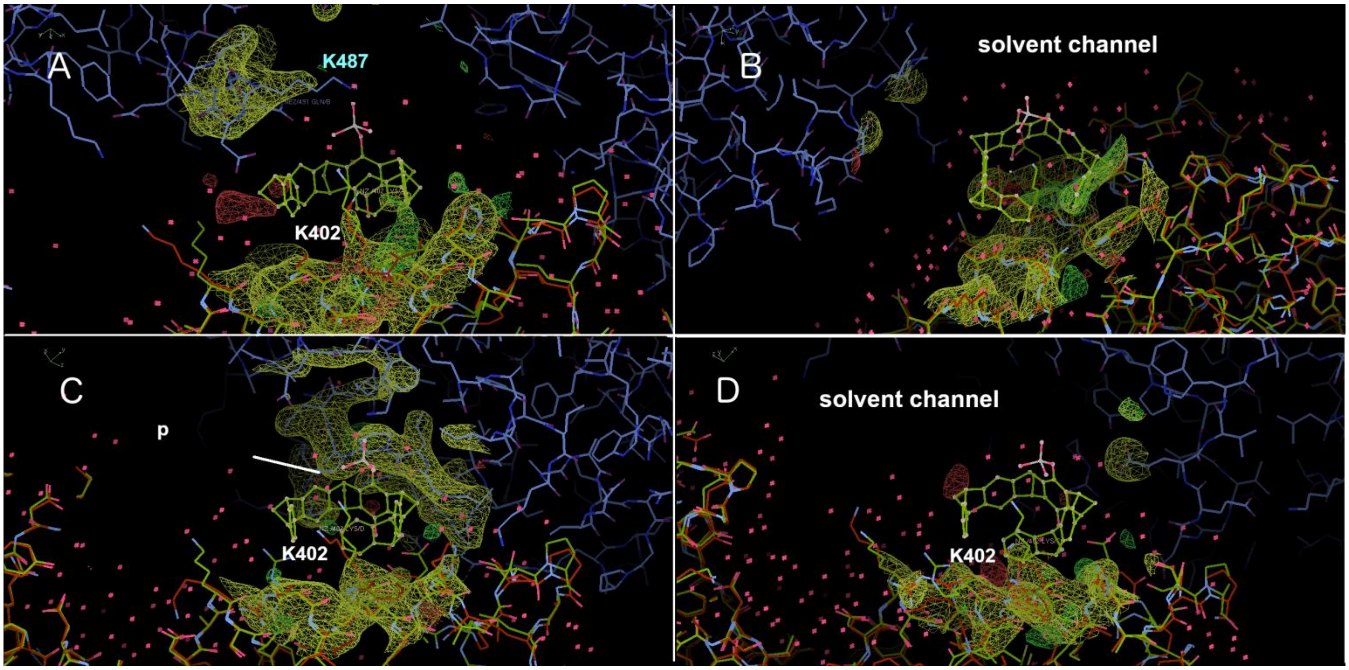

Supplementary Figure S5:

Environment of Lys402 in the $P6_5$ crystal form of P5CDH (red sticks with yellow electron density map), including the symmetry related molecules (shown in blue). The crystal packing potentially allows binding of a monophosphate-tweezer (MPT) molecule on residue Lys402 in three of four monomers, so this crystal form would have been compatible with MPT binding. The reason for the formation of a different crystal form could be either the different crystallization condition or the induction of a more favourable crystal packing by the MPT molecule sitting in the interface between two P5CDH monomers.
